# Supplementary material for: Sympathetic axonal sprouting induces changes in macrophage populations and protects against pancreatic cancer
Source: Nat Commun. 2022 Apr 13;13:1985. doi: 10.1038/s41467-022-29659-w (PMC9007988; doi:10.1038/s41467-022-29659-w)
Supplement: Supplementary file 1 — Supplementary Information [file 41467_2022_29659_MOESM1_ESM.pdf]

Supplementary Information

Sympathetic axonal sprouting induces changes in macrophage populations and protects against pancreatic cancer

Jérémy Guillot[1*], Chloé Dominici[1*], Adrien Lucchesi[1*], Huyen Thi Trang Nguyen[1,2*], Angélique Puget[1], Mélanie Hocine[1], Martha M. Rangel-Sosa[1], Milesa Simic[3], Jérémy Nigri[4], Fabienne Guillaumond[4], Martin Bigonnet[4], Nelson Dusetti[4], Jimmy Perrot[5], Jonathan Lopez [6,7,8], Anders Etzerodt[3,9], Toby Lawrence[3], Pierre Pudlo[10], Florence Hubert[10], Jean-Yves Scoazec[11], Serge A. van de Pavert[3], Richard Tomasini[4], Sophie Chauvet[1#] and Fanny Mann[1#].

Supplementary Figures and Figure Legends 1-10

Supplementary Tables 1 and 2

Supplementary Reference

Supplementary Figures

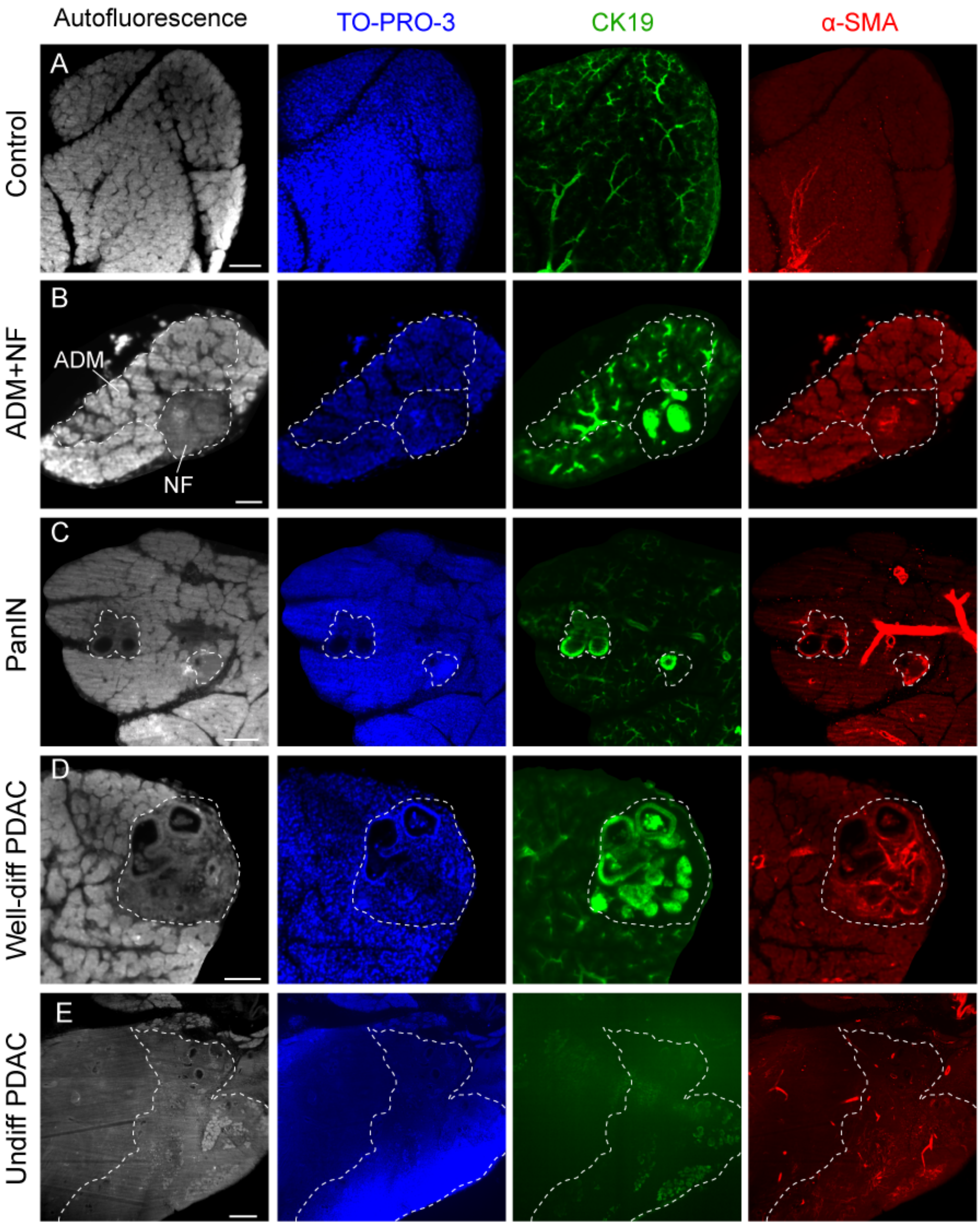

Supplementary Figure 1. Identification of regions of interest (ROI) using tissue autofluorescence. **A–E**, Optical sections through cleared pancreata of 8-week-old control (**A**) and KIC **(B–E)** mice. Images are representative of 7 mice analyzed per genotype. From left to right, panels show the acquisition of tissue autofluorescence (imaged in the blue-green spectrum with 480 nm laser excitation), nuclear counterstaining with TO-PRO-3, and immunofluorescent signals of anti-CK19 and anti-α-SMA antibodies. Images show normal acinar tissue (**A**), ADM and NF (**B**), PanIN lesions (**C**), well-differentiated PDAC (**D**), and undifferentiated/anaplastic PDAC regions (**E**). ROI are delimited by a white dashed line. In control pancreas, CK19 is expressed by epithelial cells of pancreatic ducts and α-SMA is expressed by smooth muscle cells in vessel walls. In pretumor and tumor lesions, CK19 is expressed by cancer cells and α-SMA is expressed by cancer-associated fibroblasts (CAFs). Tissue autofluorescence provides sufficient histological information to identify the different stages of disease progression. The identity of the pancreatic lesions shown above has been confirmed by an anatomopathologist. Scale bars = 50 µm (**A**, **B**), 150 µm (**C**), 100 µm (**D**), and 300 µm (**E**).

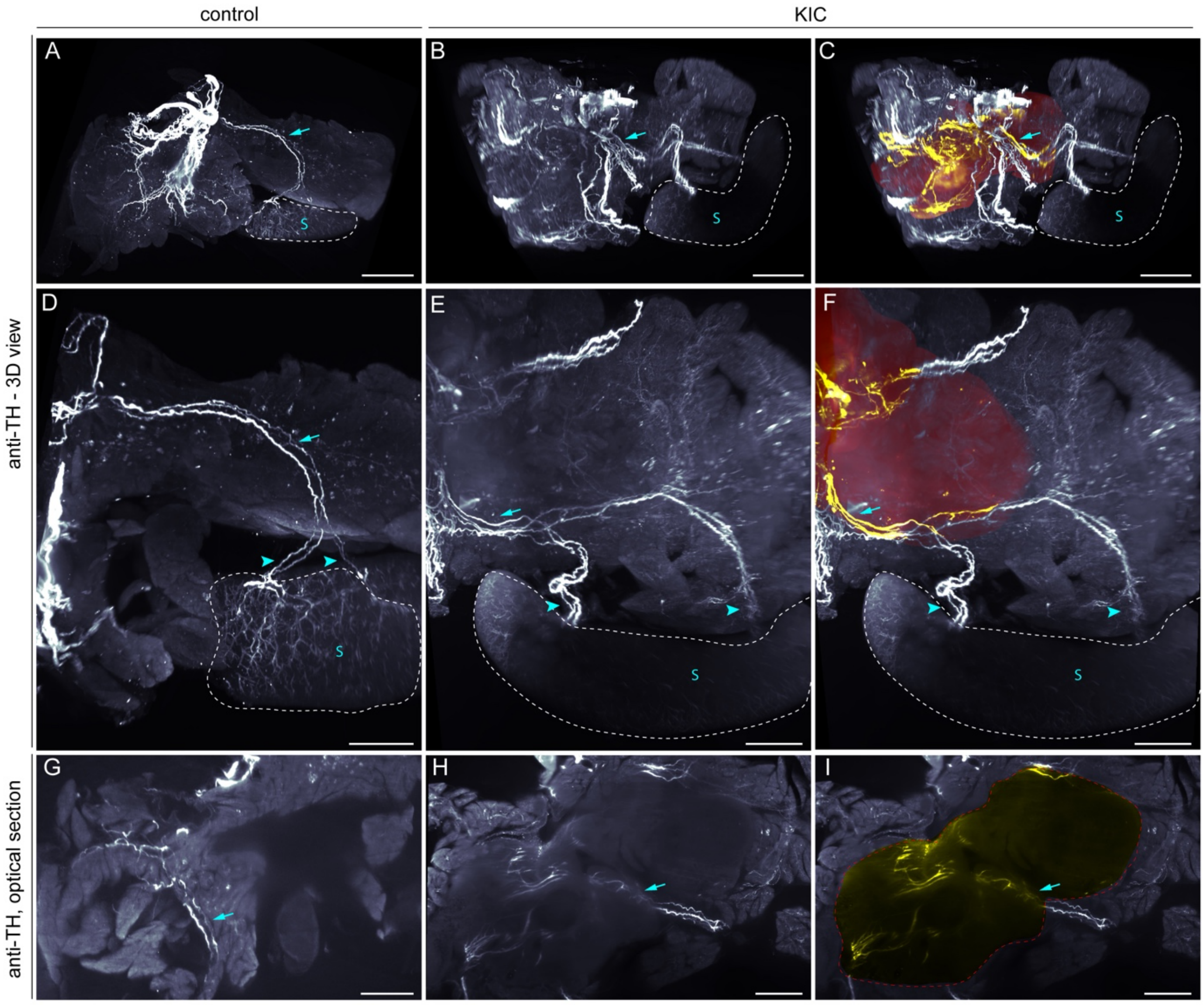

Supplementary Figure 2. 3D patterns of sympathetic nerve fiber bundles in control and KIC pancreata. Representative 3D views (**A–F**) and optical sections (**G–I**) of anti-TH labeled pancreas and spleen (s, delimited with dotted line) of 8-week-old control (**A, D**, and **G**) and KIC (**B**, **C**, **E**, **F**, **H**, and **I**) mice. All panels are LSFM images of solvent-cleared tissues and are representative of 4 mice analyzed for each genotype. The blue arrow points to the splenic nerve, whose branches (arrowheads) project to the spleen. **C, F, I,** 3D segmentation of pancreatic tumor (red), intra-tumoral TH^+ nerve fibers (yellow), and extra-tumoral TH^+ nerve fibers (white). Scale bars = 2 mm (**A**), 3 mm (**B**, **C**), and 1 mm (**D-I**).

A IMAGE TREATMENT

Imaris tools		Extracted data
**Surface**	→	- *Volume* of ROI
**Surface**	→	- *Volume* of duct lumen
**Filament tracer**	→	- *Dendrite Length* (sum)<br>- *Dendrite Length* (mean)<br>- *Dendrite Straightness* (mean)<br>- *Filament No. Dendrite Branch Pts*<br>- *Filament No. Sholl Intersections*
**Surface**	→	- *Volume* of blood vessels
**Surface-Surface Contact Area**	→	- *% SurfaceArea coverage*<br>- *Contact surface area*<br>- *Number of Disconnected Components*<br>- *Disconnected Components area (mean)*

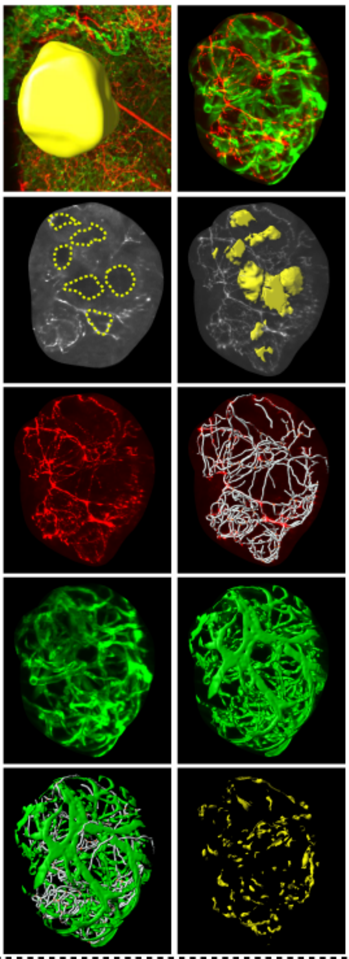

B DATA ANALYSIS

AXONAL NETWORK

Paramater	Unit
Axon density	nm / µm³
Branching	branch points / mm
Branch lenght	µm
Branch straightness	-
Branching pattern	-

AXON / BV INTERACTION

Paramater	Unit
% Contact axon / BV	%
Contact size	µm²
Contact fragmentation	contacts / µm²
Contact density	contacts / µm³
BV density	-
% Contact BV / axon	%
Contact / Volume BV	contacts / µm³

C STATISTICAL ANALYSIS

→ Z-score → PCA

Supplementary Figure 3. Image processing and quantitative analysis of local remodeling of sympathetic axons. A, LSFM images were processed with Imaris software. ROI were identified via tissue autofluorescence (imaged at 488 nm excitation). A mask was created around each ROI using the "Surface" tool, after which the ROI volume was collected in the statistics tab. In non-invasive pancreatic lesions and PDAC, masks were created around the lumen of epithelial lesions using the "Surface" tool and the volume of the lumen (empty space) was collected and deduced from the ROI volume (see also Supplementary Fig. 4). Axonal networks were reconstructed manually using "Filament tracer." The following parameters were collected in the statistics tab: dendrite length (sum and mean values), defined as the length of each axon branch; dendrite straightness (mean value), defined as the ratio between branch length and radial distance between two branch points; filament no. Sholl intersections, defined as the number of branch intersections per concentric spheres originating at the centroid of the ROI; and filament no. dendrite branch pts, defined as the number of branching points in the entire axon network. The "Surface" tool was used to reconstruct vascular networks and collect the volume occupied by the blood vessels. Finally, the MATLAB plug-in Imaris XTension "Surface-Surface contact area" was used to generate the contact surface areas between axonal and blood vessels networks. In the statistic tab, we collected the number of disconnected components, defined as the number of contacts between the two surfaces; the disconnected components area (mean value), defined as the area of each contact region; and the contact surface area; defined as the total surface of contacts. We then collected the % Surface Area coverage, defined as the percentage of axon surface in contact with blood vessels (% contact axon/BV) or as the percentage of blood vessel surface in contact with axons (% contact BV/axon). **B**, the extracted data were used to calculate 12 parameters describing the morphology of axonal networks and interactions between axons and blood vessels (BV). Details of the calculations are provided in Supplementary Table 1. Statistical analysis was performed using MATLAB and R software (**C**).

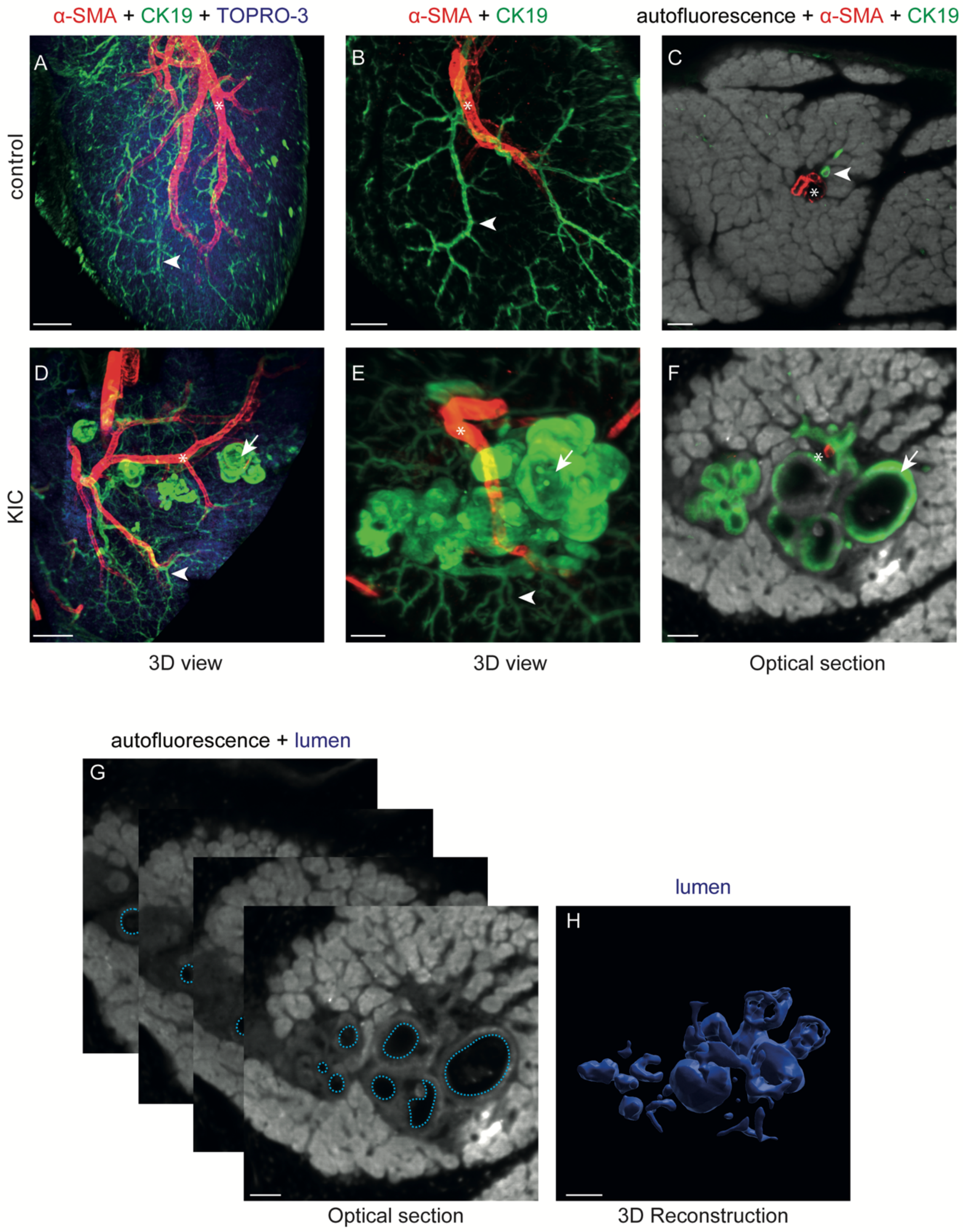

Supplementary Figure 4. 3D visualization of pancreatic epithelial lesions and lumen reconstruction.

Maximal intensity projection of 3D volume (**A-B**, **D-E**) and optical sections (**C** and **F)** of anti-α-SMA and anti-CK19 labeled pancreatic lobules of 8-week-old control (**A-C**) and KIC (**D-F**) mice. Asterisks indicate

blood vessels; arrowheads indicate pancreatic ducts and arrows point to epithelial lesions. All panels are LSFM images of solvent-cleared tissues and are representative of 7 mice analyzed for each genotype. **G** and **H**, the lumen of the epithelial lesions present in KIC pancreas were segmented on optical sections images of tissue autofluorescence (imaged at 488 nm excitation) or CK19 staining (**G)** and their volume reconstructed (**H**). Scale bars = 100 µm (**A**, **D**), 50 µm (**B**), 25 µm (**C, E, F, G**), and 20 µm (**H**).

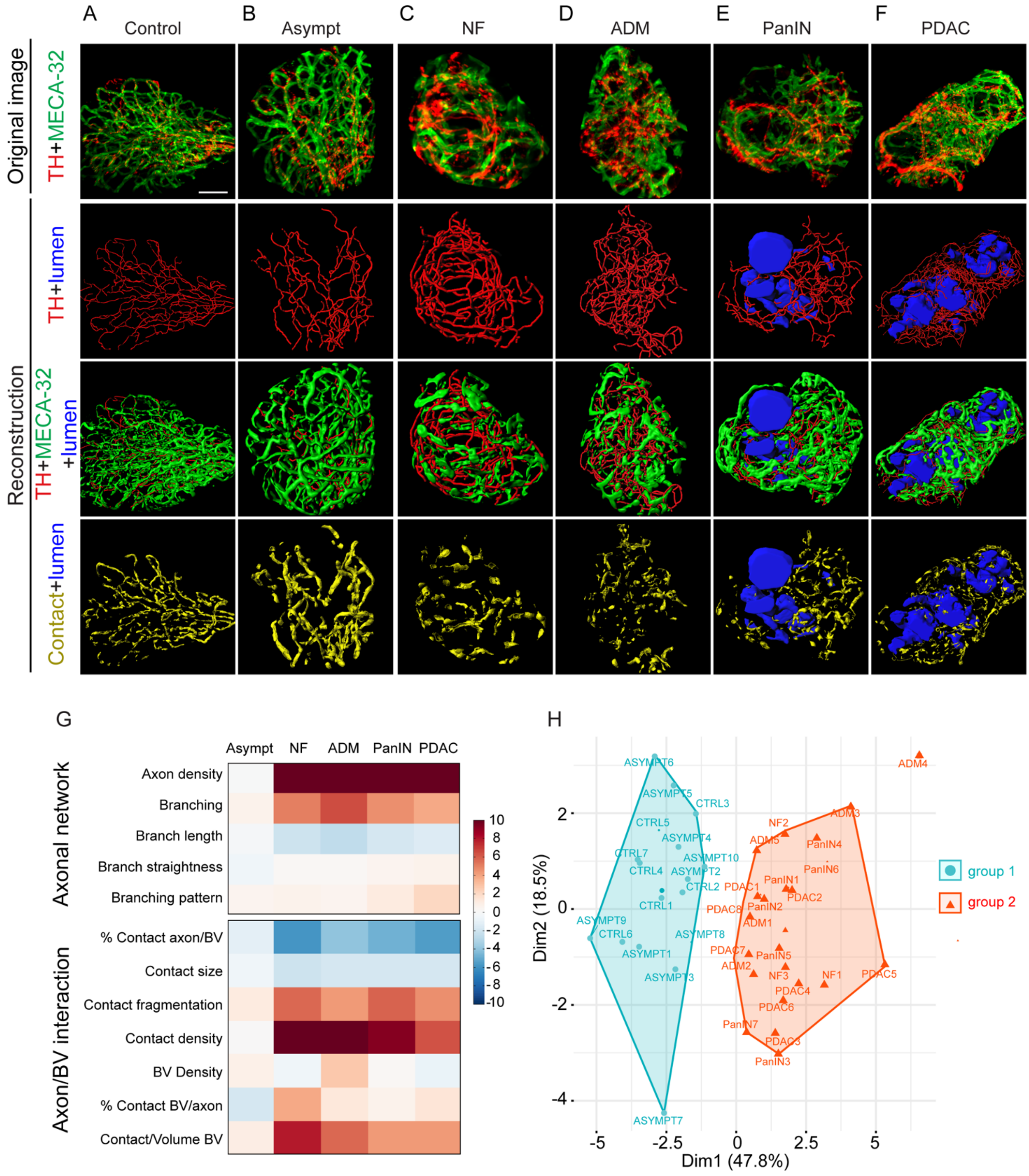

Supplementary Figure 5. 3D visualization and statistical analysis of sympathetic axon and blood vessel networks in KPC pancreas.

A–F, Representative images of 14-week-old control (**A**) or KPC (**B–F**) pancreata immunostained with anti-TH and MECA-32 antibodies (first row). 3D reconstructions of sympathetic axons (red, second row), blood vessels (green, third row), and axon/vessel surface contacts (yellow, fourth row) in normal acinar tissue (**A**), asymptomatic acinar tissue (**B**), NF (**C**), ADM (**D**), PanIN (**E**), and a well-differentiated PDAC region (**F**). Lumen of the epithelial lesions are represented in blue. Images are representative of 3 control and 4 KPC mice analyzed. **G,** Heatmap of the Z-scores calculated for each of the 12 variables describing the architecture of sympathetic axons and their relationship with blood vessels. Values in 10 asymptomatic acinar regions (Asymp), 3 NF, 5 ADM, 7 PanIN, and 8 PDAC samples of 14-week-old KPC mice (n = 4) were compared with those of 7 normal acinar regions in age-matched control mice (n = 3). **H,** Factor map of the PCA performed on 40 tissue samples and 12 variables describing sympathetic innervation. Two cluster groups were identified corresponding to control and asymptomatic tissues (group 1, blue) and pre-tumor and tumor lesions (group 2, red). Scale bars = 25 µm (**C**), 50 µm (**A**, **B** and **D–F**). Source data are provided as a Source Data file.

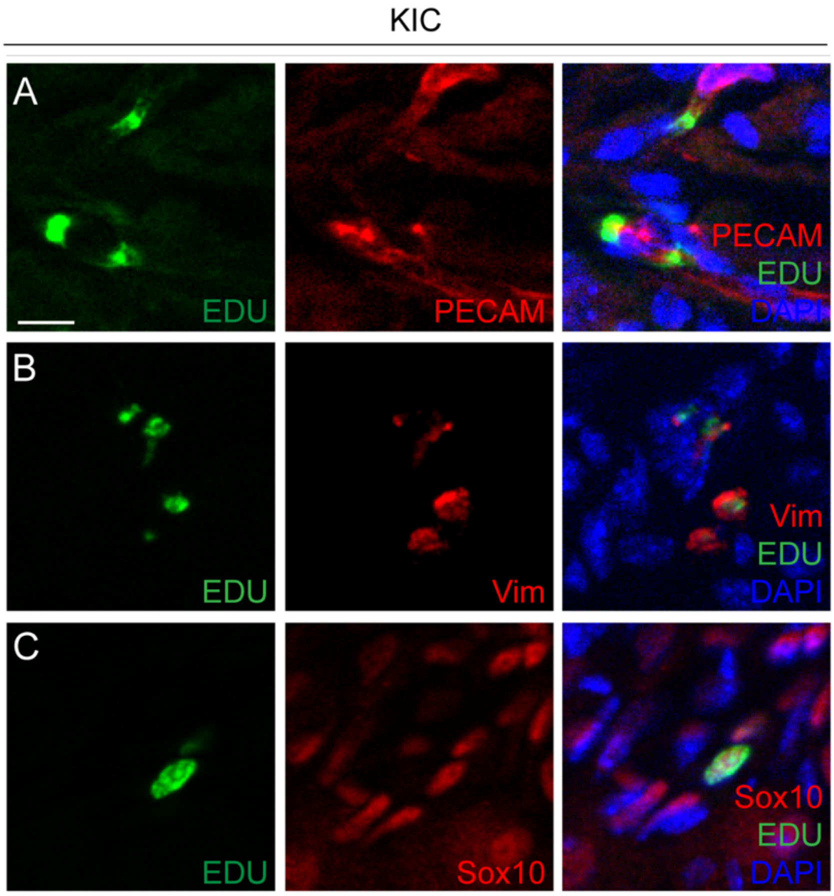

Supplementary Figure 6. Identity of EdU+ cells in the coeliac-superior mesenteric ganglia of KIC mice

A–C, representative images of EDU^{+} cells in the coeliac-superior mesenteric complex of 5.5 -week-old KIC mice, immunostained with anti-PECAM (endothelial cell marker, **A**), anti-Vimentin (fibroblasts marker, **B**) and anti-Sox10 (glial cells marker, **C**). Images are representative of 3 mice analyzed.

Scale bars= 20 µm

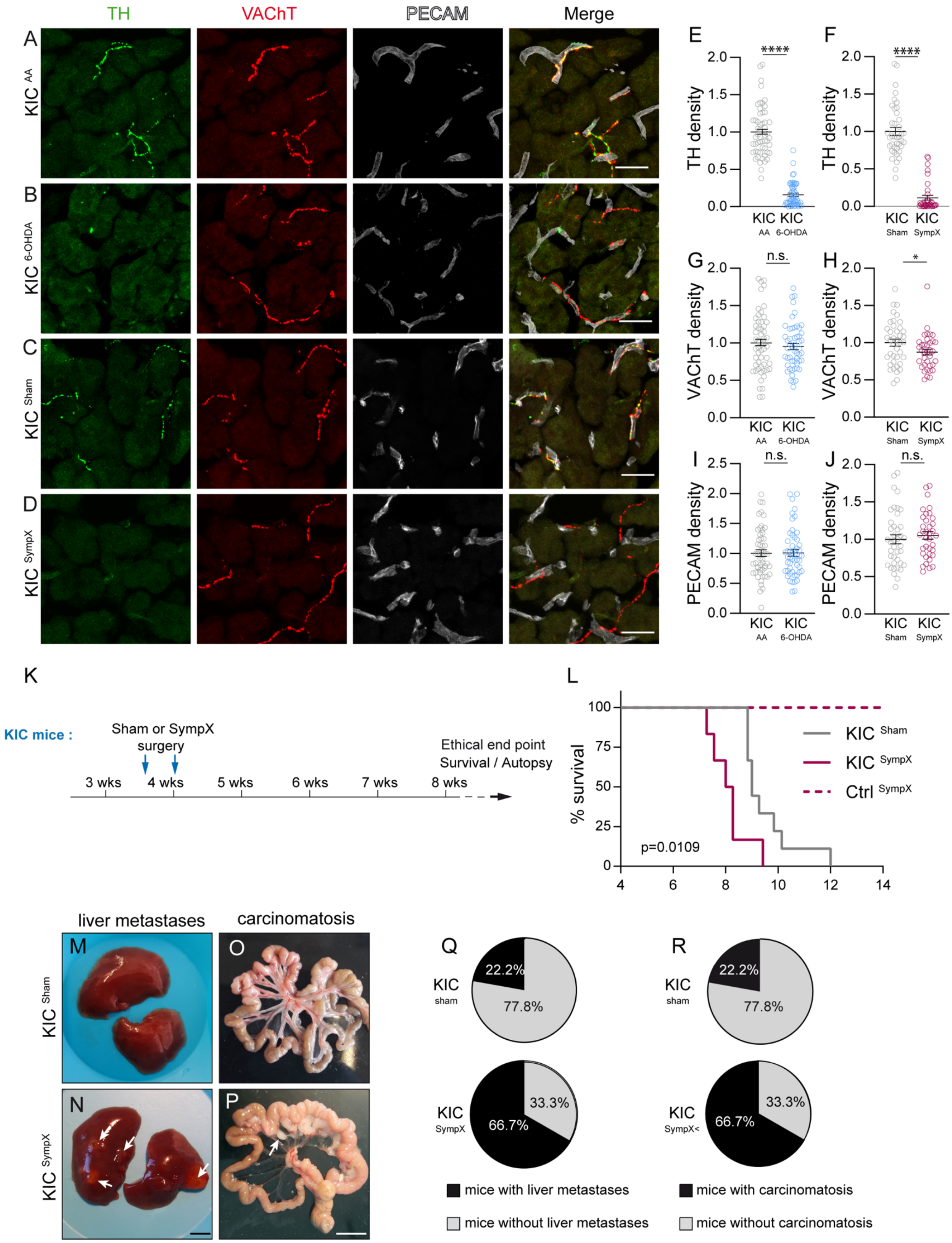
TH
VAChT
PECAM
Merge
A
KIC AA
B
KIC 6-OHDA
C
KIC Sham
D
KIC SympX
E
TH density
****
KIC AA
KIC 6-OHDA
F
****
KIC Sham
KIC SympX
G
VAChT density
n.s.
H
*
I
PECAM density
n.s.
J
n.s.
K
KIC mice :
Sham or SympX
surgery
Ethical end point
Survival / Autopsy
3 wks
4 wks
5 wks
6 wks
7 wks
8 wks
L
% survival
KIC Sham
KIC SympX
Ctrl SympX
p=0.0109
liver metastases
carcinomatosis
M
O
N
P
KIC Sham
KIC SympX
Q
R
KIC sham
22.2%
77.8%
KIC SympX
66.7%
33.3%
KIC SympX<
mice with liver metastases
mice without liver metastases
mice with carcinomatosis
mice without carcinomatosis

Supplementary Figure 7. Effects of sympathectomy on tumor growth, metastatic spread, and survival.

A–D, Representative images of pancreatic acinar tissue immunonostained with anti-TH, anti-VAChT, and anti-PECAM antibodies. The pancreas was collected from mice treated with AA (**A**) or 6-OHDA (**B**) or from mice that had undergone sham surgery (Sham; **C**) or surgical sympatectectomy (SympX; **D**) two weeks after chemical or surgical sympathectomy. **E–J,** Histograms representing the density of TH^{+} fibers (**E, F**), $VAChT^{+}$ fibers (**G, H**), and $PECAM^{+}$ vessels (**I, J**) in the pancreas of sympathectomized mice shown in (**A-D**). Data are presented as mean ± SEM; $p < 0.0001$ (**E, F**), $p = 0.5$ (**G**) and $p = 0.0423$ (**H**) with Mann–Whitney test; $p = 0.96$ (**I**) and $p = 0.52$ (**J**) with unpaired t-test. KIC^{AA}, n = 2 mice, 54 images; $KIC^{6\text{-}OHDA}$, n = 2 mice, 52 images; KIC^{Sham}, n = 2 mice, 40 images; KIC^{SympX}, n = 2, 36 images. **K,** Outline of the in vivo experiments. **L,** Kaplan–Meier curve comparing overall survival of KIC mice with intact pancreas (KIC^{Sham}, n = 9) or with surgical sympathectomy (KIC^{SympX}, n = 6) and control sympathectomized mice ($Ctrl^{SympX}$, n = 5). KIC^{Sham} vs KIC^{SympX}, $p = 0.0109$ (Log-rank test) and hazard ratio B/A = 3.179 (A = KIC^{Sham} and B = KIC^{SympX}). **M–P,** Representative pictures of livers and intestine of KIC^{Sham} and KIC^{SympX} mice collected at autopsy. **Q–R,** Pie charts showing the percentage of KIC^{Sham} (n = 6) and KIC^{SympX} (n = 9) mice with or without liver metastasis (**Q**) or carcinomatosis in the intestine mesentery (**R**) at death. Scale bars = 50 µm (**A–D**), 5 mm (**M, N**). Source data are provided as a Source Data file.

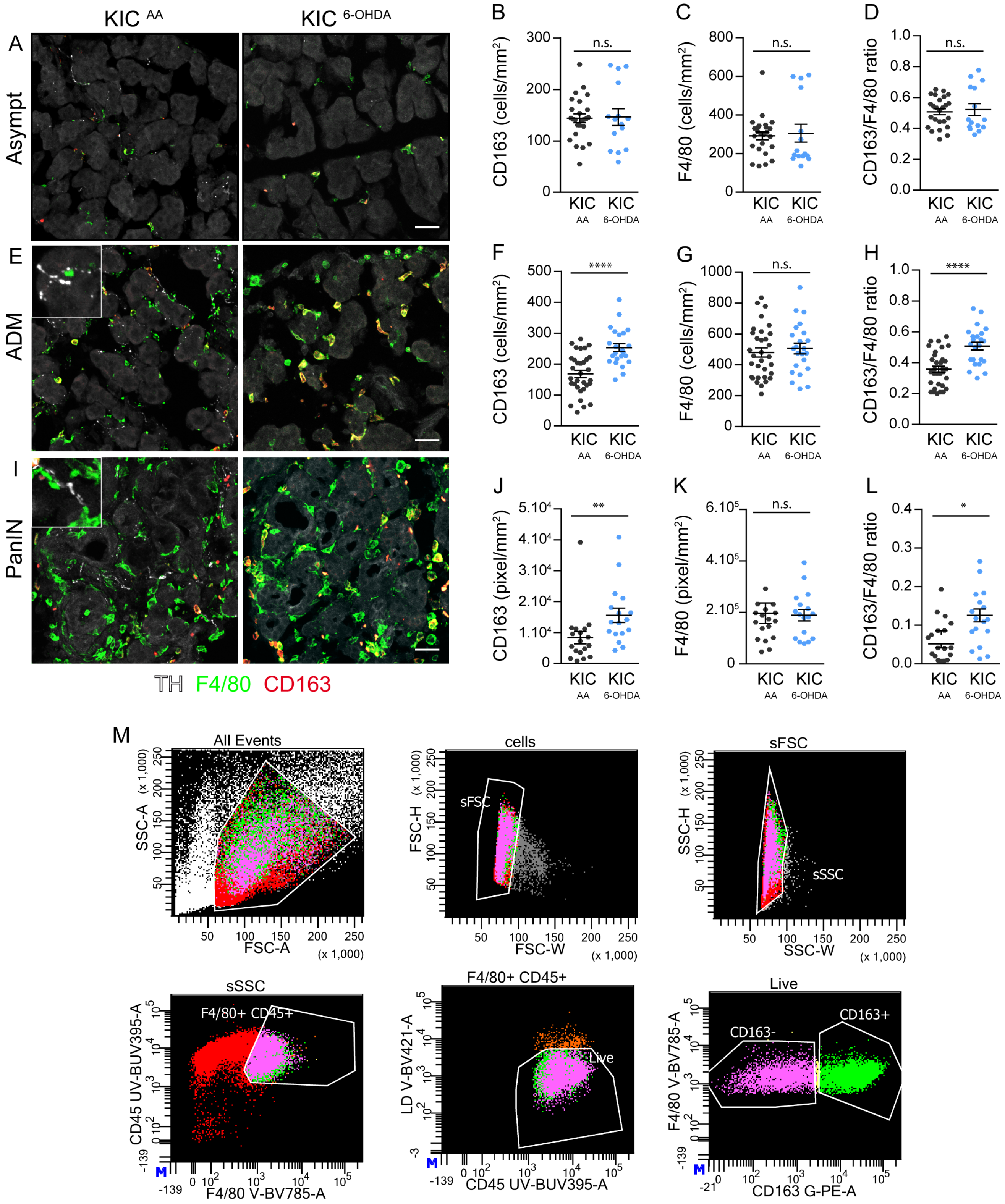

N

Tube: Pancreas

Population	#Events	%Parent	%Total
All Events	50,000	####	100.0
cells	30,543	61.1	61.1
sFSC	29,462	96.5	58.9
sSSC	29,244	99.3	58.5
F4/80+ CD45+	8,173	27.9	16.3
Live	7,816	95.6	15.6
CD163+	4,677	59.8	9.4
CD163-	3,009	38.5	6.0

Supplementary Figure 8. Distribution and FACS sorting of CD163+ pancreatic macrophages.

A, Immunostaining for F4/80, CD163, and TH in asymptomatic acinar tissue (Asympt) of 6.5-week-old KIC mice treated with AA or 6-OHDA. **B–D**, Scattered dot plots depicting the density of CD163+ macrophages (**B**; $p = 0.7720$, Mann–Whitney test), F4/80+ macrophages (**C**; $p = 0.3411$, Mann–Whitney test), and CD163/F4/80 ratio (**D**; $p = 0.9956$, Mann–Whitney test) in asymptomatic regions of AA and 6-OHDA-treated KIC mice. KIC^{AA}, n = 4 mice, 25 images; $KIC^{6\text{-}OHDA}$, n = 3 mice, 15 images. **E**, Immunostaining for F4/80, CD163, and TH in ADM of 6.5-week-old KIC mice treated with AA or 6-OHDA. **F–H**, Scattered dot plots depicting the density of CD163+ macrophages (**F**; $p < 0.0001$, unpaired *t*-test), F4/80+ macrophages (**G**; $p = 0.5667$, unpaired *t*-test), and CD163/F4/80 ratio (**H**; $p < 0.0001$, Mann–Whitney test) in the ADM of AA- and 6-OHDA-treated KIC mice. KIC^{AA}, n = 6 mice, 33 images; $KIC^{6\text{-}OHDA}$, n = 4 mice, 23 images. **I**, Immunostaining for F4/80, CD163, and TH in PanIN lesions of 6.5-week-old KIC mice treated with AA or 6-OHDA. **J–L**, Scattered dot plots depicting the density of CD163+ macrophages (**J**; $p < 0.0001$, Mann–Whitney test), F4/80+ macrophages (**K**; $p = 0.0024$; Mann–Whitney test), and CD163/F4/80 ratio (**L**; $p = 0.0153$, Mann–Whitney test) in PanIN of AA- and 6-OHDA-treated KIC mice. KIC^{AA}, n = 4 mice, 18 images; $KIC^{6\text{-}OHDA}$, n = 4 mice, 17 images. Data are presented as median ± SEM. **M,** Representative FACS gating strategy for the isolation of $CD163^{+}$ macrophages from mouse pancreas. Briefly, cells were gated according to size and granularity, with doublets subsequently removed. Macrophages were gated as CD45+/F4/80+ cells and live cells were gated inside this population. Finally, CD163+ cells were selected for use in experiments shown in Fig. 10H. **N**, Gating hierarchy of a representative sample. Scale bars= 50 µm. Source data are provided as a Source Data file.

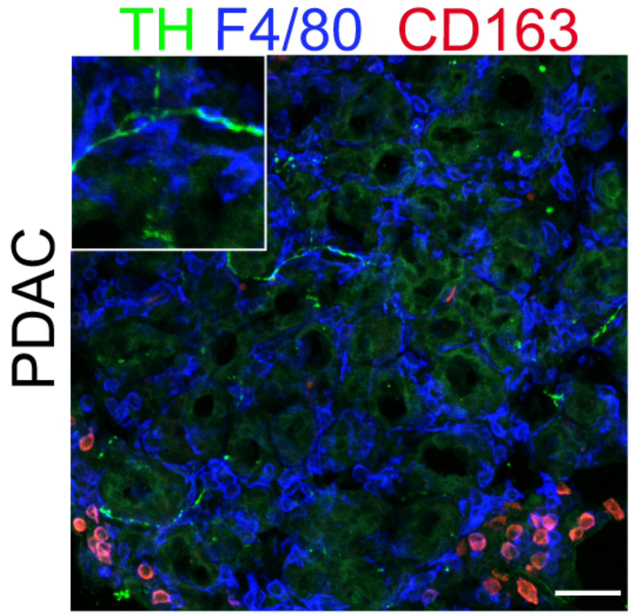

Supplementary Figure 9. Proximity between macrophages and the sympathetic innervation of PDAC tissues.

Immunostaining for F4/80, CD163, and TH in PDAC tissue of a 6.5-week-old KIC mouse. Images are representative of 4 mice analyzed. Scale bar = 50 µm.

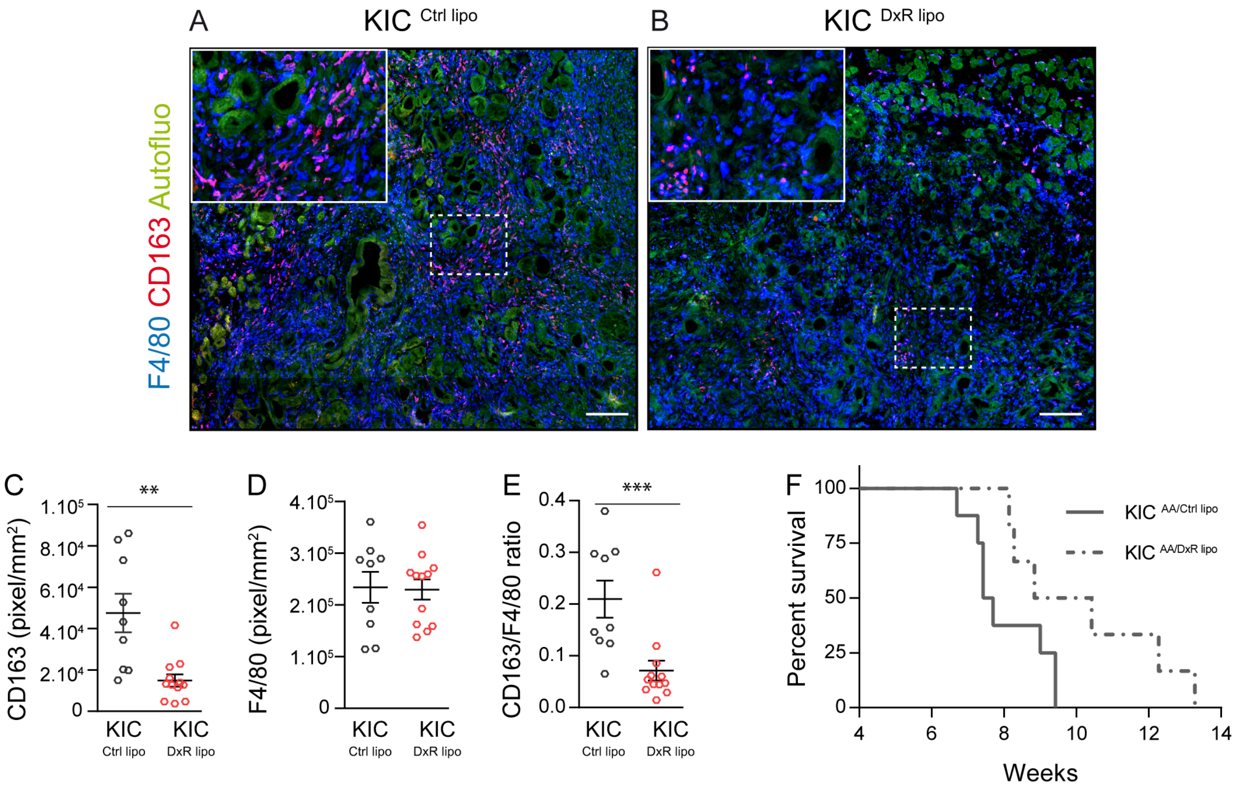

Supplementary Figure 10. Validation of CD163+ macrophage ablation.

A and **B,** Immunolabeling with anti-F4/80 and anti-CD163 antibodies of PDAC sections from 6.5-week-old KIC mice injected twice a week with Ctrl lipo or DxR lipo for 3 weeks. Tissue histology is revealed via autofluorescence. **C–F,** Scattered dot plots depicting the density of CD163+ macrophages (**C**; p = 0.0013, Mann–Whitney test), F4/80+ macrophages (**D**; p = 0.9001, unpaired *t*-test), and CD163/F4/80 ratio (**E**; p = 0.0003, Mann–Whitney test) in PDAC of 6.5-week-old KIC mice treated for 3 weeks with Ctrl lipo ($KIC^{Ctrl\ lipo}$, n = 2 mice, 9 images) or DxR lipo ($KIC^{DxR\ lipo}$, n = 3 mice, 12 images). Data are presented as median ± SEM. **F**, Kaplan–Meier curve comparing overall survival of AA-treated KIC mice injected with Ctrl lipo ($KIC^{AA/Ctrl\ lipo}$, n = 8, same as in Figure 10L) or DxR lipo ($KIC^{AA/DxR\ lipo}$, n = 8). $KIC^{AA/Ctrl\ lipo}$ vs. $KIC^{AA/DxR\ lipo}$, p=0.0109 (Log-rank test) and hazard ratio B/A = 3.665 (A = $KIC^{AA/Ctrl\ lipo}$ and B = KIC $^{6-AA/DxR\ lipo}$). Scale bars= 200 µm. Source data are provided as a Source Data file.

Supplementary Table 1: Definition of parameters describing axonal morphology and relationships with blood vessels

Parameters	Algorithm	Units
Axon density	$\frac{\mathit{Dendrite\ length}\ \text{(sum)}\ X\ 1000}{\mathit{Volume}\ \text{of the ROI} - \sum \mathit{Volume}\ \text{of the cavities}}$	nm / μm^3
Branching	$\frac{\mathit{Filament\ No.Dendrite\ Branch\ Pts}\ X\ 1000}{\mathit{Dendrite\ length}\ (\mathit{Sum})}$	branch points / mm
Branch length	*Dendrite Length* (mean)	µm
Branch straightness	*Dendrite Straightness* (mean)	--
Branching pattern	*Filament No. Sholl Intersections*	--
% contacts axon/BV	*% SurfaceArea coverage*<br>(primary surface = surface of filaments)	%
Contact size	*Disconnected Components area* (mean)	μm^2
Contact fragmentation	$\frac{\mathit{Number\ of\ Disconnected\ Components}}{\mathit{Contact\ surface\ area}}$	contacts /μm^2
Contact density	$\frac{\mathit{Number\ of\ Disconnected\ Components}}{\mathit{Volume}\ \text{of the ROI} - \sum \mathit{Volume}\ \text{of the cavities}}$	contacts/ μm^3
BV density	$\frac{\mathit{Volume}\ \text{of the BV}}{\mathit{Volume}\ \text{of the ROI} - \sum \mathit{Volume}\ \text{of the cavities}}$	--
% contact BV/axon	% SurfaceArea coverage<br>(primary surface = vascular surface)	%
Contact/Volume BV	$\frac{\mathit{Number\ of\ Disconnected\ Components}}{\mathit{Volume}\ \text{of the BVs}}$	contacts /μm^3

Supplementary Table 2: Referential list of antibodies used for immunohistochemistry

Antibody name	Host animal	Dilution	Source	Identifier
**Primary antibodies**				
Anti-Tyrosine Hydroxylase (TH) antibody	rabbit	1:200	Abcam	Cat # ab76442, RRID: AB_1524535
Anti-Tyrosine Hydroxylase (TH) antibody	chicken	1:200	Aves Labs	Cat # TYH, RRID: AB_10013440
Anti-Tyrosine Hydroxylase (TH) antibody	rabbit	1:200	Merck	Cat# AB152, RRID: AB_390204
Anti-Vesicular Acethylcholine Transporter (VAChT) antibody	rabbit	1:250	Synaptic Systems	Cat # 139 103, RRID:AB_887864
Anti- CD31 Platelet Endothelial Cell Adhesion Molecule (PECAM) antibody	rat	1:400	BD BioScience	Cat # 553370, RRID: AB_394816
Anti-Synaptophysin 1 antibody	guinea pig	1:400	Synaptic Systems	Cat #101 004, RRID: AB_1210382
Anti-F4/80 antibody	rat	1:50	Santa Cruz Biotechnology	Cat # sc-71088, RRID: AB_1122714
Anti-CD163 antibody	rabbit	1:1000	non-commercial	(1)
Anti-CD163 (M-96) antibody	rabbit	1:100	Santa Cruz Biotechnology	Cat # sc-33560, RRID : AB_2074556
Anti-CD45 antibody	rat	1:1000	Thermo Fisher Scientific	Cat # 13-0451-82, RRID: AB_466446
Anti-Ki-67 antibody	mouse	1:400	BD Biosciences	Cat # 550609, RRID: AB_393778
Anti-Actin smooth muscle (□-SMA) antibody	rabbit	1:300	Abcam	Cat # ab5694, RRID: AB_2223021
Anti-Actin smooth muscle (□-SMA) antibody	goat	1:1500	Novus	Cat# NB300-978, RRID: AB_2273630
Anti-Mouse cytokeratin 19 (CK-19) antibody	rat	1:40	DSHB	Cat # TROMA-III, RRID: AB_2133570
Anti-Panendothelial Cell Antigen Monoclonal Antibody, Clone MECA-32	rat	1:10	BD Biosciences	Cat # 550563, RRID: AB_393754
Anti-Insulin antibody	rabbit	1:200	Cell Signaling Technology	Cat # 4590, RRID : AB_659820
Anti-Insulin antibody	guinea pig	1:2	Agilent	Cat# IR002, RRID: AB_2800361
Anti-Doublecortin (DCX) antibody	rabbit	1:500	Abcam	Cat # ab18723, RRID: AB_732011
Anti-Sox10 antibody	rabbit	1:800	Abcam	Cat # ab155279, RRID : AB_2650603
Anti-Vimentin antibody, Clone Vim-13.2	mouse	1:200	Sigma-Aldrich	Cat# V5255, RRID : AB_477625
**Secondary antibodies**				
Alexa Fluor 568-conjugated anti-Chicken IgY (H+L) antibody	goat	1:500	Thermo Fisher Scientific	Cat# A-11041, RRID: AB_2534098

Cy3- conjugated anti-Guinea Pig IgG (H+L) antibody	donkey	1:500	Jackson Immuno Research Labs	Cat# 706-165-148, RRID: AB_2340460
Alexa Fluor 488-AffiniPure Donkey Anti-Guinea Pig IgG (H+L) (min X Bov,Ck,Gt,Sy Hms,Hrs,Hu,Ms,Rb,Rat,Shp Sr Prot) antibody	donkey	1:500	Jackson Immuno Research Labs	Cat# 706-545-148, RRID: AB_2340472
Alexa Fluor 488-conjugated anti-Rabbit IgG (H+L) antibody	donkey	1:500	Thermo Fisher Scientific	Cat# A-21206, RRID: AB_2535792
Cy3-conjugated anti-Rabbit IgG (H+L) antibody	donkey	1:500	Jackson Immuno Research Labs	Cat# 711-165-152, RRID: AB_2307443
Alexa Fluor 568-conjugated anti-Rabbit IgG (H+L) antibody	donkey	1:500	Thermo Fisher Scientific	Cat#A10042, RRID: AB_2534017
Alexa Fluor 647- conjugated anti-Rabbit IgG (H+L) antibody	donkey	1:500	Thermo Fisher Scientific	Cat# A-31573, RRID: AB_2536183
Alexa Fluor 790-conjugated anti-Rabbit IgG (H+L) antibody	donkey	1:500	Jackson Immuno Research Labs	Cat# 711-655-152, RRID: AB_2340628
Alexa Fluor 647-conjugated anti-Rat IgG (H+L) antibody	donkey	1:500	Jackson Immuno Research Labs	Cat#712-605-153, RRID: AB_2340694
Alexa Fluor 790-conjugated anti-Rat IgG (H+L) antibody	donkey	1:500	Jackson Immuno Research Labs	Cat# 712-655-153, RRID: AB_2340701
Cy3-conjugated anti-Rat IgG (H+L) antibody	donkey	1:500	Jackson Immuno Research Labs	Cat# 712-165-153, RRID: AB_2340667
Alexa Fluor 647-conjugated anti-Goat IgG (H+L) antibody	donkey	1:500	Thermo Fisher Scientific	Cat# A-21447, RRID: AB_2535864
Alexa Fluor 488-conjugated anti-Mouse IgM mu chain antibody	donkey	1:500	Abcam	Cat# ab150121, RRID: AB_2801490

Supplementary reference

1. Etzerodt A, Moestrup SK. CD163 and inflammation: Biological, diagnostic, and therapeutic aspects. Antioxidants Redox Signal. 2013. page 2352–63.
