# Supplementary material for: Sympathetic axonal sprouting induces changes in macrophage populations and protects against pancreatic cancer
Source: Nat Commun. 2022 Apr 13;13:1985. doi: 10.1038/s41467-022-29659-w (PMC9007988; doi:10.1038/s41467-022-29659-w)
Supplement: Supplementary file 2 — Reporting Summary [file 41467_2022_29659_MOESM2_ESM.pdf]

natureresearch

Corresponding author(s): Fanny MANN

Last updated by author(s): Mar 21, 2022

Reporting Summary

Nature Research wishes to improve the reproducibility of the work that we publish. This form provides structure for consistency and transparency in reporting. For further information on Nature Research policies, see Authors & Referees and the Editorial Policy Checklist .

Statistics

For all statistical analyses, confirm that the following items are present in the figure legend, table legend, main text, or Methods section.

n/a	Confirmed	
☐	☒	The exact sample size (*n*) for each experimental group/condition, given as a discrete number and unit of measurement
☐	☒	A statement on whether measurements were taken from distinct samples or whether the same sample was measured repeatedly
☐	☒	The statistical test(s) used AND whether they are one- or two-sided<br>*Only common tests should be described solely by name; describe more complex techniques in the Methods section.*
☒	☐	A description of all covariates tested
☐	☒	A description of any assumptions or corrections, such as tests of normality and adjustment for multiple comparisons
☐	☒	A full description of the statistical parameters including central tendency (e.g. means) or other basic estimates (e.g. regression coefficient) AND variation (e.g. standard deviation) or associated estimates of uncertainty (e.g. confidence intervals)
☐	☒	For null hypothesis testing, the test statistic (e.g. *F*, *t*, *r*) with confidence intervals, effect sizes, degrees of freedom and *P* value noted<br>*Give P values as exact values whenever suitable.*
☐	☒	For Bayesian analysis, information on the choice of priors and Markov chain Monte Carlo settings
☒	☐	For hierarchical and complex designs, identification of the appropriate level for tests and full reporting of outcomes
☐	☒	Estimates of effect sizes (e.g. Cohen's *d*, Pearson's *r*), indicating how they were calculated

Our web collection on statistics for biologists contains articles on many of the points above.

Software and code

Policy information about availability of computer code

Data collection	Image data were acquired with Zeiss Axio Zoom V16, Zeiss Lumar V12, Zeiss Axio Imager 2, Zeiss Axio Imager M2 with Apotome , Zeiss LSM 780 , Zeiss LSM 880 with Airyscan, Miltenyi Biotec Ultramicroscope II and Biospace Lab Photon Imager.
Data analysis	The data were analyzed with Fiji, ImageJ (v_1.51m9), Excel, Graphpad Prism (v_6 & v_9), Imaris x64 (v_9.2.1 & v_9.3.0), Calopix, BD FACSDiva v_9.0.1, Stan with NUTS sampling algorithm, R and Matlab (R2014b MCR v8.4).<br>The code used to perform the bioluminescence data analysis in Fig. 9E-9I of the article is available at:<br>https://github.com/pierrepudlo/SASmacrophage

For manuscripts utilizing custom algorithms or software that are central to the research but not yet described in published literature, software must be made available to editors/reviewers. We strongly encourage code deposition in a community repository (e.g. GitHub). See the Nature Research guidelines for submitting code & software for further information.

Data

Policy information about availability of data

All manuscripts must include a data availability statement. This statement should provide the following information, where applicable:

- Accession codes, unique identifiers, or web links for publicly available datasets
- A list of figures that have associated raw data
- A description of any restrictions on data availability

The data generated in this study are available within the manuscript and the Supplementary materials. Source data are provided with this paper. Full microscopy image data sets are available upon request.

Field-specific reporting

Please select the one below that is the best fit for your research. If you are not sure, read the appropriate sections before making your selection.

- [x] Life sciences
- [ ] Behavioural & social sciences
- [ ] Ecological, evolutionary & environmental sciences

For a reference copy of the document with all sections, see nature.com/documents/nr-reporting-summary-flat.pdf

Life sciences study design

All studies must disclose on these points even when the disclosure is negative.

Sample size	Sample sizes are provided in the figure legend. Sample size was calculated by power analysis using BiostaTGV (http://biostatgv.sentiweb.fr) for survival studies and measurement of total nerve volume. The power was set at 90% and the level of significance at 5%. No statistical methods were used to pre-determine sample sizes in the other experiments, but sample sizes are similar to those generally employed in the field.
Data exclusions	No samples or animals were excluded.
Replication	Each experiment was replicated a minimum of three times and data was reliably reproduced with each replication attempt.
Randomization	Mice were genotyped for allocation into control (Cre negative) or tumor (KIC (LSL-KrasG12D/+; Ink4a/Arflox/lox; Pdx1-Cre) and KPC (LSL-KrasG12D/+; LSL-P53R172H/+; Pdx1-Cre)) groups. All animals were randomly divided into control and treatment groups.
Blinding	Animals were genotyped before the experiments, i.e., no blinding was used to allocate the experimental groups. Collection of 3D image data and analysis of control versus tumor tissue was not performed: indeed, the presence or absence of a tumor is obvious, therefore blinding is not possible and not relevant in this situation. Analyses of tumor samples that received different treatments were performed in a blinded fashion: in this situation the tissues cannot be distinguished, blinding is therefore relevant and was applied.

Reporting for specific materials, systems and methods

We require information from authors about some types of materials, experimental systems and methods used in many studies. Here, indicate whether each material, system or method listed is relevant to your study. If you are not sure if a list item applies to your research, read the appropriate section before selecting a response.

Materials & experimental systems

n/a	Involved in the study	
[ ]	[x]	Antibodies
[ ]	[x]	Eukaryotic cell lines
[x]	[ ]	Palaeontology
[ ]	[x]	Animals and other organisms
[ ]	[x]	Human research participants
[x]	[ ]	Clinical data

Methods

n/a	Involved in the study	
[x]	[ ]	ChIP-seq
[ ]	[x]	Flow cytometry
[x]	[ ]	MRI-based neuroimaging

Antibodies

Antibodies used	A referential list of antibodies used is given in Supplementary Table 3.
Validation	We used only previously published and validated or commercial antibodies.<br>Chicken anti-Tyrosine Hydroxylase antibody: manufacturer validation (https://www.abcam.com/tyrosine-hydroxylase-antibody-ab76442.html). Hammond, S.L. et al PLoS One. 2017 Dec 15;12(12):e0188830. doi: 10.1371/journal.pone.0188830. Tested by IF and WB on mouse tissue.<br>Rabbit anti-Tyrosine Hydroxylase antibody: manufacturer validation (https://www.aveslabs.com/products/tyrosine-hydroxylase-tyh). Cho JR, et al Neuron. Published: June 08, 2017 DOI:https://doi.org/10.1016/j.neuron.2017.05.020. Tested by IF on mouse tissue.<br>Anti-Vesicular Acethylcholine Transporter antibody: manufacturer validation.(https://www.sysy.com/products/vacht/facts-139103.php). Cutuli D et al., Int J Mol Sci. Published online 2020 Mar 4. doi: 10.3390/ijms21051741. Tested by IF on mouse tissue.<br>Anti-CD31 antibody : manufacturer validation (https://www.bdbiosciences.com/eu/applications/research/stem-cell-research/cancer-research/mouse/purified-rat-anti-mouse-cd31-mec-133/p/553370). Duncan et al., J Immunol 1999, 162:3022-3030; Tested by IHC on mouse tissue.<br>Anti-Synaptophysin 1 antibody: manufacturer validation. (https://www.sysy.com/products/s-physin1/facts-101004.php). Gerbino et al., Neuron. Published: March 27, 2020 DOI: https://doi.org/10.1016/j.neuron.2020.03.005. Tested by IF on mouse tissue.<br>Anti-F4/80 antibody: (http://datasheets.scbt.com/sc-71088.pdf). Zhang et al, World J Gastroenterol. 2016 Mar 14;22(10):2960-70. doi: 10.3748/wjg.v22.i10.2960. Tested by IHC on mouse tissue.

Anti-CD163 antibody: Etzerodt et al., J Exp Med. 2020 Apr 6;217(4). pii: e20191869. doi: 10.1084/jem.20191869. Tested by IF on mouse tissue.
Anti-CD163 (M96) antibody: Rubio-Navarro et al., Theranostics. 2016 Apr 21;6(6):896-914. doi: 10.7150/thno.14915. eCollection 2016. Tested by IF on human tissue.
Anti-CD45 antibody: manufacturer validation (https://www.thermofisher.com/antibody/product/CD45-Antibody-clone-30-F11-Monoclonal/13-0451-82). Fukui et al., J Ophtalmol. Published online 2017 Sep 30. doi: 10.1155/2017/1639012. Tested by IF on mouse tissue.
Anti-Ki-67 antibody: manufacturer validation (https://www.bdbiosciences.com/us/applications/research/intracellular-flow/intracellular-antibodies-and-isotype-controls/anti-rat-antibodies/purified-mouse-anti-ki-67-b56/p/550609). Spargo et al., International immunology. Advance Access publication 18 April 2006. doi:10.1093/intimm/dxl026. Test by IF on mouse tissue.
Anti-Actin smooth muscle antibody: manufacturer validation (https://www.abcam.com/alpha-smooth-muscle-actin-antibody-ab5694.html). Gu et al., European Journal of Pharmacology. 2018. https://doi.org/10.1016/j.ejphar.2018.10.008. Tested by IHC on mouse tissue.
Anti-Actin smooth muscle antibody: munufacturer validation (https://www.novusbio.com/products/alpha-smooth-muscle-actin-antibody_nb300-978).
Anti-Cytokeratin 19 antibody: manufacturer validation (https://scicrunch.org/resolver/AB_2133570). Loebermann et al, 2009. doi:10.1111/j.1365-3156.2009.02387.x. Tested by IHC on mouse tissue.
Anti-Panendothelial Cell antibody: manufacturer validation.(https://www.bdbiosciences.com/us/reagents/research/antibodies-buffers/immunology-reagents/anti-mouse-antibodies/cell-surface-antigens/purified-rat-anti-mouse-panendothelial-cell-antigen-meca-32/p/550563). Hallmann et al., Developmental dynamics (1995). Tested by IHC on mouse tissue.
Anti-Insulin antibody: manufacturer validation (https://www.cellsignal.com/products/primary-antibodies/insulin-antibody/4590). Liu et al., Frontiers in Endocrinology. 2020. doi: 10.3389/fendo.2020.00032. Tested by IHC on mouse tissue.
Anti-Insulin antibody: manufacturer validation (https://www.agilent.com/en/product/immunohistochemistry/antibodies-controls/primary-antibodies/insulin-(autostainer-link-48)-76277). Alvarsson et al. Sci. Adv. 6, eaaz9124 (2020). doi: 10.1126/sciadv.aaz9124. Tested by IF on cleared mouse pancreas.
Anti-Doublecortin antibody: manufacturer validation (https://www.abcam.com/doublecortin-antibody-ab18723.html). Starossom et al., Jan 15;10(1):217. doi: 10.1038/s41467-018-08140-7. Tested by IF on mouse tissue.
Anti-Sox10 antibody: manufacturer validation (https://www.abcam.com/sox10-antibody-epr4007-ab155279.html). Tested by IF on mouse tissue.
Anti-Vimentin antibody: manufacturer validation (https://www.sigmaaldrich.com/FR/en/product/sigma/v5255).

Eukaryotic cell lines

Policy information about cell lines

Cell line source(s)	The PK4A-Luc cell line (RRID:CVCL_WB21) was obtained from Fabienne Guillaumond (CRCM, Marseille, France). R211-Luc cells were generated by Pierre Cordelier (CRCT, Toulouse, France) and are R211 cells (from Dieter Sauer, TUM, Munich, Germany) modified to express red-shifted, firefly luciferase (RSLucF).
Authentication	The cell line were authenticated by PCR analysis.
Mycoplasma contamination	All cell lines were tested negative for micoplasma.
Commonly misidentified lines (See ICLAC register)	This study did not use cell lines listed in ICLAC database.

Animals and other organisms

Policy information about studies involving animals; ARRIVE guidelines recommended for reporting animal research

Laboratory animals	Mus Muculus, C57BL/6 background or a mixed FVB/C57BL/6 background, both sexes, 3 to 14-week-old.<br>KIC (LSL-KrasG12D/+; Ink4a/Arf(Cdkn2a)lox/lox; Pdx1-Cre), both sexes, 3.5 to 8-week-old.<br>KPC (LSL-KrasG12D/+; LSL-Trp53R172H/+; Pdx1-Cre), both sexes, 14-week-old.<br>Animals were housed under controlled conditions (12 h light/dark cycle; humidity 45–65%; room temperature 23±2 °C) and provided ad libitum access to water and food.
Wild animals	This study did not involve wild animals.
Field-collected samples	This study did not involve field-collected samples.
Ethics oversight	All animal procedures were conducted in accordance with the guidelines of the French Ministry of Agriculture (approval number F1305521) and approved by the Ethics Committee for animal experimentation of Marseille – CEEA-014 (approval number APAFIS#1325-2016120211301815v1, APAFIS#17278-2018102514126851 V3, and APAFIS#17026-2018092610362806 v6).

Note that full information on the approval of the study protocol must also be provided in the manuscript.

Human research participants

Policy information about studies involving human research participants

Population characteristics	Pancreatic cancer samples come from the CRO2 (Center for Research in Oncobiology and Oncopharmacology) biobank. The tissue samples were obtained after pancreatic resection in 13 female (mean age: 69 years) and 21 male (mean age: 66 years) patients, who underwent surgery without treatment. The number of participants is too small to determine whether age or gender had an influence on the results.<br>Written or verbal informed consent from participant patients was not obtained specifically for this study, because (1) only histopathological samples for which patients had consented to inclusion and storage in the biobank, according to biobank legislation, were included in the study; and (2) further specific written consent was considered unfeasible due to the retrospective nature of the study and the general poor prognosis associated with the tumor type in this study.
Recruitment	Participants were patients with a proven diagnosis of PDAC, operable from the start, without preoperative treatment or comorbidities.
Ethics oversight	Regional Ethical Review Board of CRO2 - Center for Research in Oncobiology and Oncopharmacology, Marseille, France (approval number DC-2013-1857)

Note that full information on the approval of the study protocol must also be provided in the manuscript.

Flow Cytometry

Plots

Confirm that:

- [x] The axis labels state the marker and fluorochrome used (e.g. CD4-FITC).
- [x] The axis scales are clearly visible. Include numbers along axes only for bottom left plot of group (a 'group' is an analysis of identical markers).
- [x] All plots are contour plots with outliers or pseudocolor plots.
- [x] A numerical value for number of cells or percentage (with statistics) is provided.

Methodology

Sample preparation	Wild-type C57BL/6 mice (n=3) were transcardially perfused with PBS. Pancreata were immediately collected and dissociated using Collagenase I (4000 U; cat. #17018-029; GIBCO), hyaluronidase type I-S (4000 U; cat. #H3506; Sigma-Aldrich), DNase I (4000 U; cat. #11284932001; Sigma-Aldrich), and 500 µL HEPES in 10 mL final volume of RPMI medium for 1 h at 37 °C with agitation. Single cell suspensions were enriched for CD45+ population using CD45 magnetic microbeads (cat. #130052301; Miltenyi Biotec) on the autoMACS Pro separator. Cells were blocked in 15 % normal mouse serum (cat. #015-000-120; Jackson Immunoresearch) and subsequently stained with CD45-BUV395 (clone 30-F11; cat. # 564279; BD Biosciences; RRID:AB_2651134), F4/80-BV785 (clone BM8; cat. #BLE123141; Biolegend; RRID:AB_2563667) and CD163-PE (clone TNKUPJ; cat. #12-1631-82; Thermofisher/Life technologies; RRID:AB_2716924). LIVE/DEAD™ Fixable Violet Dead Cell (cat. # L34955; Thermofisher) was used for viability staining.
Instrument	Cell sorting was performed with the BD FACS ARIA III cell sorter
Software	Flow cytometry data were analyzed with BD FACSDiva 9.0.1
Cell population abundance	Abundance of the relevant cell populations within post-sort fractions is detailed in Supplementary Fig. 8N.
Gating strategy	The gating strategy is illustrated in Supplementary Fig. 8M. Using the FSC/SSC gating, cells were gated according to size and granularity, with doublets subsequently removed. Macrophages were gated as CD45+/F4/80+ cells and live cells were gated inside this population. Finally, CD163+ cells were selected.

- [x] Tick this box to confirm that a figure exemplifying the gating strategy is provided in the Supplementary Information.
