# Supplementary material for: Sympathetic axonal sprouting induces changes in macrophage populations and protects against pancreatic cancer
Source: Nat Commun. 2022 Apr 13;13:1985. doi: 10.1038/s41467-022-29659-w (PMC9007988; doi:10.1038/s41467-022-29659-w)
Supplement: Supplementary file 3 — Description of Additional Supplementary Files [file 41467_2022_29659_MOESM3_ESM.pdf]

Title: Supplementary Movie 1 :
Description: 3D visualization of a whole pancreas from an 8-week-old control mouse immunostained with an anti-TH antibody to reveal sympathetic innervation.

Title: Supplementary Movie 2 :
Description: 3D visualization of a whole pancreas from an 8-week-old KIC mouse immunostained with an anti-TH antibody to reveal sympathetic innervation.

Title: Supplementary Data 1 :
Description: Data analysis of axon morphology and interactions with blood vessels in KIC and KPC pancreata

Title: Supplementary Data 2 :
Description: DSP counts and data analysis
